# Medical Examiner

**Published:** 1845-03

**Authors:** 


*Medical Examiner.*—This excellent Medical Periodical is now published monthly, instead of every two weeks, as formerly. Each number of the old series contained twelve pages, and each number of the new, seventy-two; but, notwithstanding it has been greatly enlarged, and improved in appearance, it is furnished to subscribers at the same price as formerly, three dollars per annum.

To speak of the character of a Medical Journal, so extensively and favorably known as this, would be a work of supererogation on our part. The acknowledged ability with which it is edited, renders any remarks from us, upon this point, unnecessary.—*Balt. Ed.*

